# C-Terminal Extension of the Yeast Mitochondrial DNA Polymerase Determines the Balance between Synthesis and Degradation

**DOI:** 10.1371/journal.pone.0033482

**Published:** 2012-03-14

**Authors:** Katrin Viikov, Olga Jasnovidova, Tiina Tamm, Juhan Sedman

**Affiliations:** Department of Biochemistry, Institute of Molecular and Cell Biology, University of Tartu, Tartu, Estonia; St. Georges University of London, United Kingdom

## Abstract

*Saccharomyces cerevisiae* mitochondrial DNA polymerase (Mip1) contains a C-terminal extension (CTE) of 279 amino acid residues. The CTE is required for mitochondrial DNA maintenance in yeast but is absent in higher eukaryotes. Here we use recombinant Mip1 C-terminal deletion mutants to investigate functional importance of the CTE. We show that partial removal of the CTE in Mip1Δ216 results in strong preference for exonucleolytic degradation rather than DNA polymerization. This disbalance in exonuclease and polymerase activities is prominent at suboptimal dNTP concentrations and in the absence of correctly pairing nucleotide. Mip1Δ216 also displays reduced ability to synthesize DNA through double-stranded regions. Full removal of the CTE in Mip1Δ279 results in complete loss of Mip1 polymerase activity, however the mutant retains its exonuclease activity. These results allow us to propose that CTE functions as a part of Mip1 polymerase domain that stabilizes the substrate primer end at the polymerase active site, and is therefore required for efficient mitochondrial DNA replication *in vivo*.

## Introduction

The mitochondrial genome encodes essential protein subunits of the oxidative phosphorylation complexes. Faithful replication of mitochondrial DNA (mtDNA) is therefore required for the maintenance of cellular respiratory activity. Polymerase γ (pol γ) is the only mitochondrial DNA polymerase, and it is hence solely responsible for DNA synthesis during replication, repair and recombination in mitochondria [1], [2]. Based on sequence similarity, pol γ is classified to the family A of DNA polymerases, that share the conserved family specific polymerase and 3′-5′ exonuclease motifs (Pol A–C and Exo I–III) [3], [4]. Mitochondrial DNA polymerases form a distinctive group inside the family A of DNA polymerases and contain additional pol γ signature motifs γ1–γ6 [5].

Malfunctioning of the pol γ leads to mtDNA integrity defects [6], [7]. A number of pol γ mutations have been associated with severe mitochondrial dysfunction leading to a wide range of neurological and muscular diseases in human, including progressive external ophthalmoplegia and Alpers syndrome [8], [9]. Budding yeast *Saccharomyces cerevisiae* is a useful model for the studies of mtDNA metabolism, as it is able to bypass the need of respiration when grown on a fermentable carbon source. Wild-type mtDNA (rho^+^) of respiratory competent yeast cells can be largely deleted and rearranged (rho^−^) or lost altogether (rho^0^). Rho^−^ and rho^0^ yeast strains form smaller, so-called *petite* colonies due to the impairment of oxidative phosphorylation. Several human disease associated mutations of pol γ were shown to increase the frequency of *petite* colony formation in yeast [10], [11]. Therefore, yeast model has been used to evaluate the severity and the dominance of disease causing mutations *in vivo* [12]–[14].

However, multiple structural and functional differences distinguish yeast pol γ from its metazoan homologues [15], [16]. First, γ polymerases from higher eukaryotes are known to function as multimeric complexes where accessory subunits associate with the catalytic subunit to increase processivity and catalytic activity [17]–[20]. Yeast pol γ, on the other hand, functions as a single-subunit enzyme consisting of catalytic subunit Mip1 alone [15], [21]. Mip1 is a highly processive DNA polymerase and does not require accessory factors for the stimulation of its activity [15], [22], [23]. Furthermore, in contrast to its homologues in higher eukaryotes, Mip1 can displace the complementary DNA strand during synthesis through double-stranded DNA regions [15]. Most DNA polymerases require destabilization of the duplex DNA by a ssDNA binding protein (*e.g.* T7 DNA polymerase) or the unwinding activity of a helicase (*e.g.* human pol γ) for strand displacement synthesis [24]–[27]. Some family A members, such as the DNA polymerase from bacteriophage T5 and *Escherichia coli* polymerase I, have also been shown to displace the complementary strand during DNA synthesis [24], [28]. Strand displacement activity of DNA polymerases has been shown to be important for genome replication as well as repair synthesis. φ29 DNA polymerase uses its extensive strand displacement activity during rolling circle replication of the phage genome [29], [30]. Distributive strand displacement activity is required during Okazaki fragment maturation by pol δ and for DNA repair by pol β [31]–[33]. The importance of Mip1 strand displacement activity for mtDNA maintenance in yeast is unknown.

**Figure 1:**
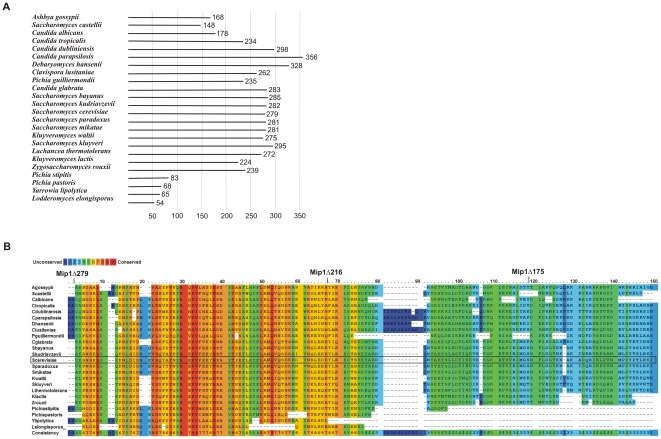
Length and amino acid sequence alignment of C-terminal extension of mitochondrial DNA polymerases from *Saccharomycetes*. A. Length of the CTE of *Saccharomycetes* species. The C-terminal extension was defined as the protein sequence starting from the 16^th^ amino acid past the γ6 motif. B. Amino acid sequence alignment of *Saccharomycetes* CTEs was performed with the PRALINE software available at www.ibi.vu.nl/programs [42] using the PAM250 weights matrix. Alignment of the first 150 residues is shown and the positions of *S. cerevisiae* Mip1Δ175, Mip1Δ216 and Mip1Δ279 deletion mutants are indicated.

Another distinctive feature of the yeast pol γ is a long C-terminal extension (CTE) that follows the γ6 motif [16]. This unique region is not present in mitochondrial DNA polymerases from higher eukaryotes, and it varies significantly in length among yeast species (Figure 1A). In *S. cerevisiae* Mip1, the CTE was shown to be required for the maintenance of mtDNA, as CTE deletion in the *mip1Δ279* mutant leads to loss of mtDNA and to respiratory incompetence [16]. Multiple sequence alignment reveals a gradient of sequence homology between CTEs from *Saccharomycetales*, with more conserved regions near the polymerase domain (Figure 1B). Based on the homology, the CTE can be subdivided into highly, moderately and poorly conserved regions [16]. In *S. cerevisiae* Mip1, the poorly conserved region covers 175 C-terminal residues or almost 2/3 of the CTE. This poorly conserved region is not essential for mtDNA maintenance as the *mip1Δ175* mutant strain forms respiratory competent colonies. [16]. Removal of the moderately conserved region of the CTE results in impaired respiratory activity in the corresponding *mip1Δ216 S. cerevisiae* strain. The *mip1Δ216* strain rapidly looses mtDNA on glucose, and displays a 3-fold reduction of total mtDNA levels as well as a 2-fold increase in doubling time on a non-fermentable carbon source [16]. BLAST searches using the fungal CTE as a query do not reveal significant similarities to any known proteins. Thus, even though the importance of the CTE for the maintenance of mtDNA integrity *in vivo* has been revealed, the biochemical function of the CTE region in the fungal mitochondrial DNA polymerase is not understood.

Here we investigate the role of the unique CTE region of yeast mitochondrial DNA polymerase using purified C-terminal deletion mutants of Mip1. Our data show that removal of the CTE leads to a complete loss of the DNA polymerase activity, explaining the rho^0^ *in vivo* phenotype. Partial deletion of the CTE, however, results in preferential exonucleolytic degradation instead of DNA synthesis, and in the reduction of strand displacement activity. This indicates that the C-terminal extension of Mip1 could function as a part of the polymerase domain that stabilizes substrate primer end at the polymerase active site.

## Results

### CTE is required for efficient DNA polymerase catalytic activity

To analyze the role of the C-terminal extension characteristic for yeast mitochondrial DNA polymerases, we constructed the C-terminal deletion mutants Mip1Δ175, Mip1Δ216 and Mip1Δ279 of the *S. cerevisiae* enzyme. Mip1Δ175 lacks the highly variable region of the CTE, Mip1Δ216 retains only the highly conserved region of the CTE and Mip1Δ279 completely lacks the CTE region (Figure 1B).

**Figure 2:**
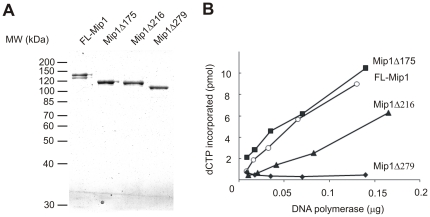
Purification and specific activity of the full-length and C-terminal deletion mutants of Mip1. A. Full-length and deletion mutants of Mip1 carrying N-terminal 6×His tag were overexpressed in *E. coli* and purified using Ni-affinity and S-Sepharose ion exchange chromatography. 0.3 µg of purified FL-Mip1 (141.0 kDa), Mip1Δ175 (120.7 kDa), Mip1Δ216 (115.9 kDa) and Mip1Δ279 (108.4 kDa) were separated on 8% SDS-PAGE. B. Specific activity of FL-Mip1 and C-terminal deletion mutants was measured by incorporation of [α -^32^P]-dCTP into calf thymus activated DNA. The activity of 8–165 ng of polymerase was measured in a 10 µl reaction in the presence of 50 µM dATP, dGTP and dTTP, 5 µM dCTP, 50 µg/ml activated calf thymus DNA and 1 µCi of [α -^32^P]-dCTP. Reaction products were spotted onto DE81 Whatman filter and washed with 0.5 M sodium-phosphate buffer pH 6.4. dCTP (pmol) incorporated in 30 min at 30°C was calculated from the percentage of incorporated radioactivity and plotted against the protein concentration: FL-Mip1 – empty circle, Mip1Δ175 –filled square, Mip1Δ216 – filled triangle, Mip1Δ279 – filled diamond.

Full-length Mip1 (FL-Mip1) and C-terminal deletion mutants were expressed in *E. coli* as previously reported [15]. Recombinant proteins lacked a mitochondrial targeting sequence of 29 amino acids and contained a 6×His tag at the protein N-terminus. An N-terminal 6×His tag was chosen to exclude possible interference of the tag with biochemical properties determined by the C-terminal region of the polymerase. FL-Mip1 and deletion mutants were purified with a combination of Ni-affinity and S-Sepharose cation exchange chromatography. The average purification yield was 0.2–0.5 mg of Mip1 per 1 L of the bacterial culture. 0.3 µg of purified FL-Mip1 and deletion mutants were analyzed on the 8% SDS-PAGE (Figure 2A). FL-Mip1 purified as a doublet, whereas the C-terminal deletion mutants of Mip1 did not display similar degradation. Thus, the two bands of FL-Mip1 formed probably due to premature translation termination or specific C-terminal degradation. The concentration of the purified proteins was established from A280 absorbance.

Specific DNA polymerase activity of the purified proteins was measured on activated calf-thymus DNA (Figure 2B). A similar nucleotide incorporation rate was recorded for FL-Mip1 and Mip1Δ175, corresponding to 66.0 and 62.4 pmol of incorporated dCTP per 1 µg of the protein in 30 min at 30°C. Mip1Δ216 displayed slightly lower incorporation rate of 45.6 pmol of dCTP per µg of the protein. No dNTP incorporation into calf-thymus activated DNA was detected for Mip1Δ279, indicating that this mutant has lost DNA polymerase activity.

**Figure 3:**
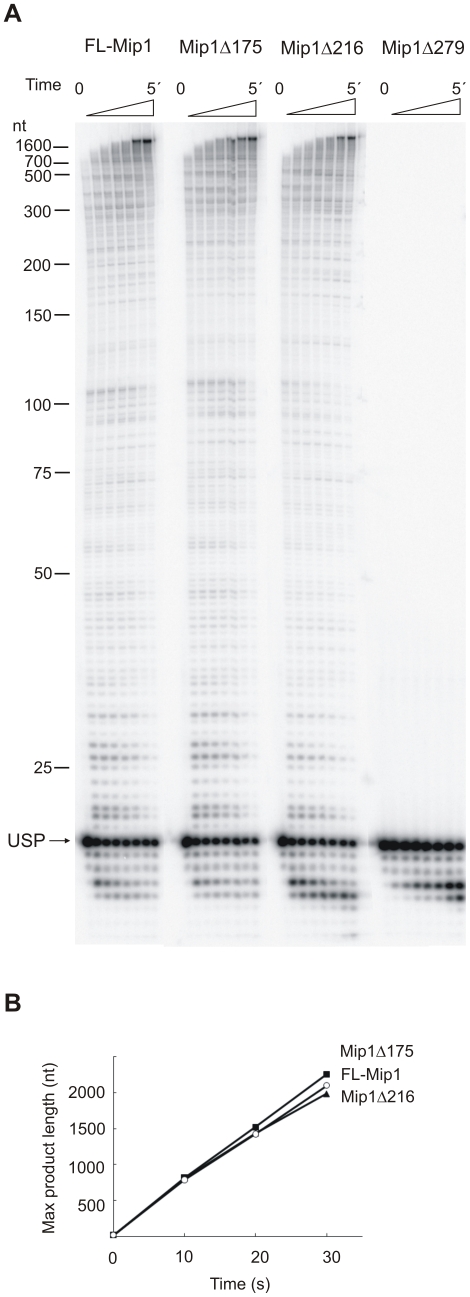
DNA polymerase activity of Mip1 and C-terminal deletion mutants. DNA polymerase activity was measured using 40 nM DNA polymerase, 4 nM M13 circular ssDNA singly primed with radiolabeled USP primer and 100 µM dNTP. The reaction was carried out at 30°C and stopped after 0 s, 10 s, 20 s, 30 s, 45 s, 1 min, 2 min and 5 min with equal volume of 80% deionized formamide, 25 mM EDTA. A. Reaction products were resolved on 8% denaturating polyacrylamide gel. The position of 17 nt USP is marked with an arrow. B. Maximum product length was determined using the DNA marker as a standard and plotted against time. FL-Mip1 – empty circle, Mip1Δ175 –filled square, Mip1Δ216 – filled triangle.

Polymerase activity of FL-Mip1 and deletion mutants was further assessed using singly primed phage M13 ssDNA (Figure 3). Elongation of the end-labeled primer was followed in time under reaction conditions where polymerase was in large molar excess over the DNA template and dNTP concentration was non-limiting (100 µM) (Figure 3A). These reaction conditions allowed determination of the maximum synthesis rate on the ssDNA. The maximum length of synthesized polymer increased linearly in time during the first 30 s of the reaction, enabling the establishment of the k_pol_ value from the slope of the line (Figure 3B). FL-Mip1, Mip1Δ175 and Mip1Δ216 displayed a similar rate of dNTP incorporation with estimated k_pol_ values of 69, 74 and 65 s^−1^, respectively (Figure 3B). Mip1Δ279 displayed no dNTP incorporation activity on singly primed M13 DNA, which is in accordance with the absence of polymerase activity on calf-thymus activated DNA. However, Mip1Δ279 did display 3′-5′ exonuclease activity as degradation of the 17 nt primer could be detected (Figure 3A).

Therefore, the polymerase assay showed that a substantial part of the CTE can be removed without affecting DNA polymerization activity of Mip1 on M13 ssDNA. Only a complete deletion of the CTE eliminating a highly conserved region of the protein results in the loss of DNA polymerase activity. Deletion of 279 amino acids from the Mip1 C-terminus probably eliminates important structural elements required for polymerase activity without affecting global folding of the Mip1 polypeptide, as the exonuclease activity is retained. The loss of polymerase activity in the Mip1Δ279 mutant apparently results in a complete loss of mtDNA, leading to respiration deficiency in *S. cerevisiae*, as previously reported [16].

### Mip1 CTE mutants support processive DNA synthesis

**Figure 4:**
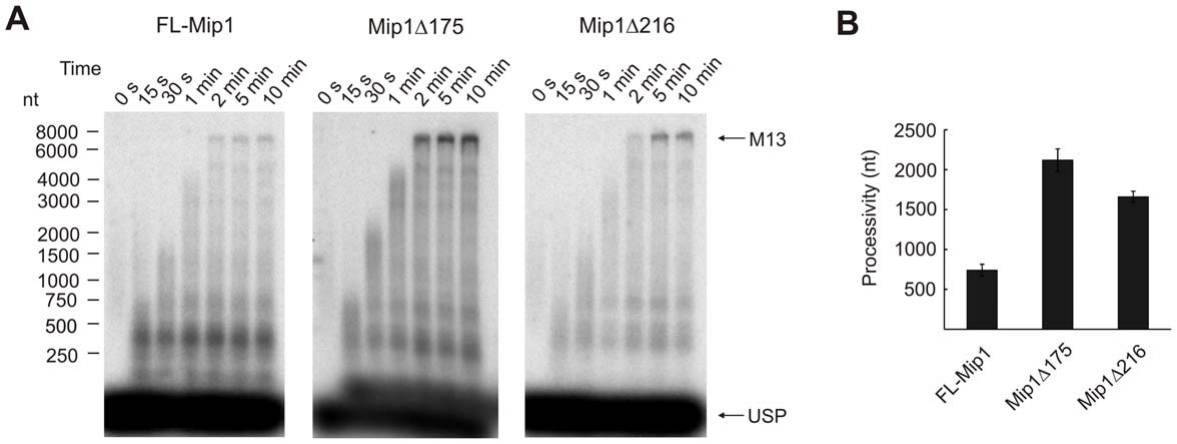
Processivity of Mip1 and C-terminal deletion mutants. Processivity was measured under single-hit conditions with 4 nM of substrate M13 ssDNA singly primed with radiolabeled USP and 1 mg/ml calf thymus activated DNA. The reaction was performed at 30°C with 4 nM DNA polymerase in the presence of 100 µM dNTP. The reaction was stopped with 0.5 mg/ml Proteinase K, 1% SDS, 20 mM EDTA after indicated time points. A. Reaction products were separated on 0.8% alkaline agarose gel. Arrows indicate positions of M13mp18 unit length (7250 nt) and 17 nt USP. B. Processivity of FL-Mip1, Mip1Δ175 and Mip1Δ216 was calculated as the average length of the product (nt) synthesized by the polymerase per one binding event. Weighted mean method based on the product intensity and length was used for analysis. Data from three independent experiments was used to calculate the average processivity and standard deviation values.

Yeast pol γ was previously reported to be a highly processive DNA polymerase [15], [22], [23]. The CTE is rich in positively charged amino acids, therefore it could be involved in establishing the high processivity of Mip1. To test this, we assessed the processivity of FL-Mip1, Mip1Δ175 and Mip1Δ216 on singly primed M13 ssDNA (Figure 4). The reaction was performed under single-hit conditions where rebinding of the polymerase to elongated substrate was prevented by large amounts of cold competitor DNA, as described in Materials and Methods. The assay enabled us to measure the processivity of the enzyme as an average number of nucleotides incorporated into the newly synthesized DNA strand before the dissociation of the polymerase from its substrate.

Elongation of the primer could be detected during the first 2–5 minutes of the processivity assay (Figure 4A). Thus, the processive DNA synthesis cycle of Mip1 lasted for up to 5 min, followed by the dissociation of the enzyme from the substrate. First reaction products corresponding to the fully elongated M13 circle (7250 nt) appeared in less than 2 min. This corresponds to a DNA synthesis rate of more than 60 nt/s. When FL-Mip1 was used, a large fraction of reaction products terminated at around 500 bp, with only a small fraction elongated to full-length M13 circle. The processivity value of FL-Mip1 on M13 DNA was estimated to be 740±70 nt from the mean length of reaction products in the 10 min time point (Figure 4B). Surprisingly, Mip1Δ175 and Mip1Δ216 displayed higher processivity than FL-Mip1. The fully elongated template was one of the dominant reaction products, and the processivity of Mip1Δ175 and Mip1Δ216 was estimated to be 2120±140 nt and 1660±70 nt, respectively.

Processivity of DNA polymerases depends on their catalytic activity and affinity for DNA. Stronger binding to the substrate DNA and a higher polymerization rate result in higher processivity of a DNA polymerase. The polymerase assay on singly primed M13 DNA revealed no differences in the dNTP incorporation activity of FL-Mip1, Mip1Δ175 or Mip1Δ216 DNA (Figure 3). Thus, the differences in processivity between FL-Mip1 and deletion mutants are probably due to their different affinity for DNA.

**Figure 5:**
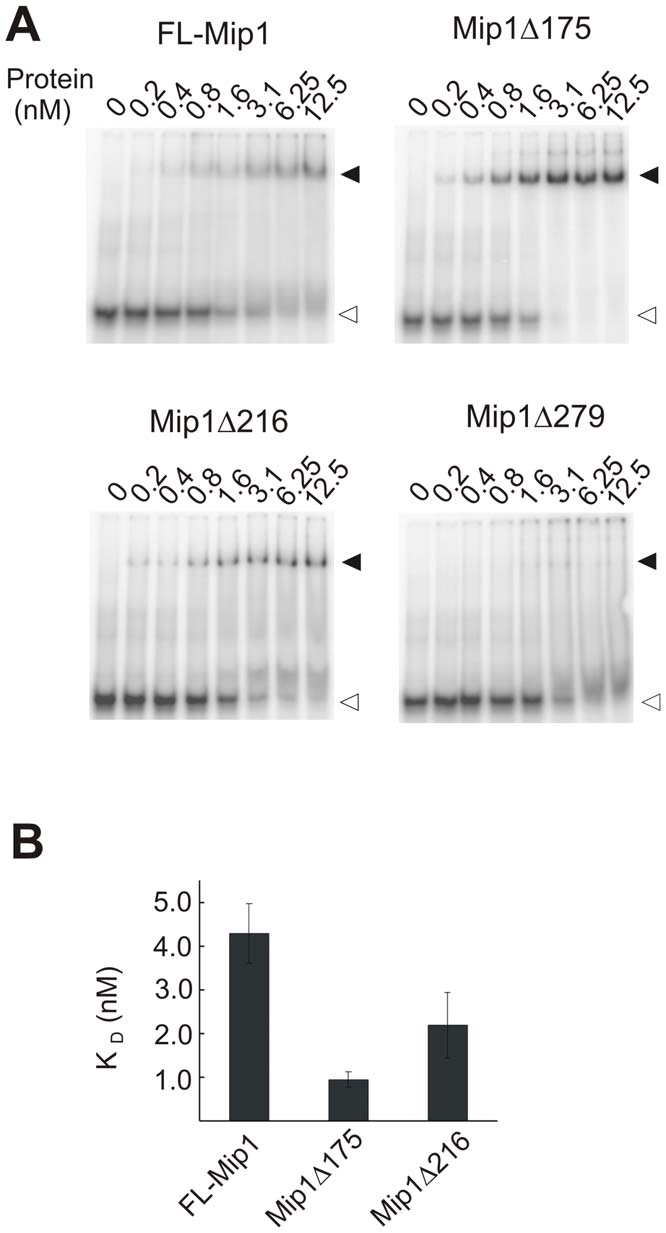
DNA binding affinity of Mip1 and C-terminal deletion mutants. DNA binding was measured with an electrophoretic mobility shift assay using an oligomeric substrate of 45 nt template strand and a radiolabeled 25 nt primer. 0.2–12.5 nM polymerase was incubated for 2 min at 0°C with 1 nM substrate. A. Reaction products were resolved on a native Tris-glycine 8% polyacrylamide gel. Positions of the free and bound substrate are indicated accordingly with empty and filled triangles. B. The dissociation constant K_D_ (nM) was calculated from the logarithmic binding curve as the concentration of the polymerase when 50% of the substrate was bound. Data from three independent experiments was used to establish the average K_D_ and standard deviation values.

To assess DNA binding affinity of FL-Mip1 and deletion mutants, we performed an electrophoretic mobility shift assay (Figure 5). Labeled 25/45 substrate was incubated with Mip1 at different protein concentrations varying from 0.2 nM to 12.5 nM. DNA/protein complex formation was detected as a shift of the labeled substrate during native gel electrophoresis (Figure 5A). A logarithmic binding curve was drawn from the ratio of the bound DNA substrate to all substrate, and dissociation constant K_D_ was estimated as the protein concentration when 50% of the substrate remained unbound (Figure 5B). The K_D_ of FL-Mip1 was estimated to be 4.3±0.68 nM. Mip1Δ175 and Mip1Δ216 both showed stronger DNA binding, with the corresponding K_D_ values estimated to 2.2±0.75 nM and 0.95±0.18 nM, respectively. Increase in DNA binding affinity correlates well with the increase of Mip1Δ175 and Mip1Δ216 processivity as compared to FL-Mip1.

Mip1Δ279 was also able to induce shift in the substrate mobility (Figure 5A). Although Mip1Δ279 bound substrate band was quite faint (filled arrow, Figure 5A), suggesting that significant amount of the protein/DNA complex was retained in the well due to possible aggregation of this mutant. For that reason we excluded Mip1Δ279 from quantitative binding affinity assessment. However Mip1Δ279 was able to bind DNA substrate as also indicated by its ability to perform exonucleolytic degradation (Figure 3A).

From these results we conclude that despite the positive charge of the CTE, this region is not required for the high DNA binding affinity and processivity of Mip1. Instead, partial removal of the CTE stimulates processivity of Mip1 by increasing its affinity towards DNA.

### Removal of CTE disturbs the balance between exonuclease and polymerase activities

**Figure 6:**
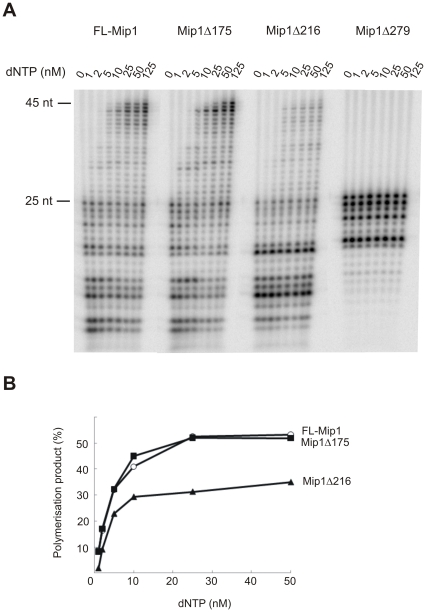
Polymerase/exonuclease balance of Mip1 and C-terminal deletion mutants. The balance between polymerase and exonuclease activities was assayed on a 45 nt template primed with a radiolabeled 25 nt primer. 4 nM DNA polymerase and 10 nM substrate were incubated for 5 min at 30°C in the presence of indicated concentrations of dNTP. Reactions were stopped with an equal volume of 80% formamide, 25 mM EDTA. A. Reaction products were resolved on 8% urea polyacrylamide gel. Positions of the 25 nt primer and 45 nt reaction product are indicated. B. The percentage of the polymerization products out of total reaction products were plotted against dNTP concentration. FL-Mip1 – empty circle, Mip1Δ175 –filled square, Mip1Δ216 – filled triangle.

Mip1 displays a potent 3′-5′ exonuclease activity that results in the accumulation of end-labeled primer degradation products. In the DNA polymerase assay on singly primed M13 DNA, the Mip1Δ216 mutant produced substantially more degraded primer products as compared to FL-Mip1 and Mip1Δ175 (Figure 3). To investigate this further, we performed exonuclease/polymerase (exo/pol) coupled assay using the 45/25 oligomeric substrate (Figure 6). When dNTPs were omitted from the reaction, no DNA polymerization occurred, and only degradation of the 25 nt primer was observed. Gradual increase of dNTP concentration in the reaction mixture shifted the balance in favor of DNA polymerization (Figure 6A).

To assess the exo/pol balance, the percentage of the polymerization products to all reaction products was calculated (Figure 6B). Mip1Δ175 displayed a shift in the exo/pol balance with increasing dNTP concentration, similar to that of FL-Mip1. At 25 nM dNTP the shift towards polymerization stabilized, with half of the reaction products subjected to polymerization. Mip1Δ216, on the other hand, displayed a significantly weaker shift from exonuclease to polymerase activity. The elongated primer constitutes only 30% of the Mip1Δ216 reaction products at 25 nM dNTP. Thus, Mip1Δ216 preferred an exonucleolytic hydrolysis over polymerization. Mip1Δ279 was also included in the analysis and displayed exonuclease activity not dependent on dNTP concentration.

**Figure 7:**
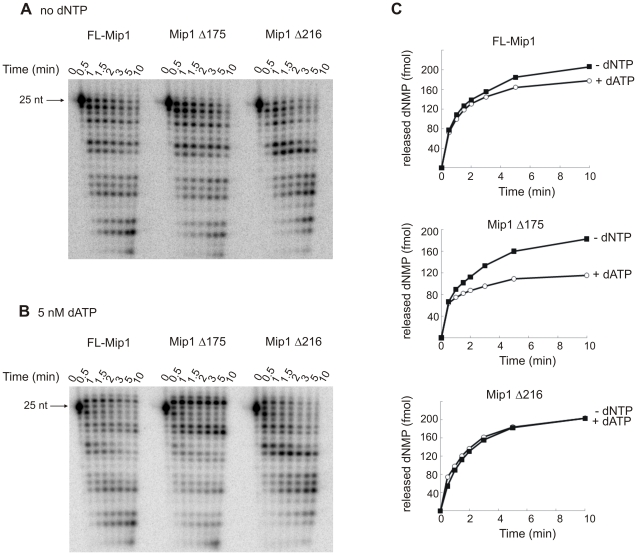
Exonuclease activity of Mip1 and C-terminal deletion mutants. Exonuclease activity was assayed on a 45 nt template primed with a radiolabeled 25 nt primer. 4 nM DNA polymerase was incubated with 2 nM 45/25 substrate in the total absence of dNTP or in the presence of 5 nM dATP. Reactions were carried out at 30°C and stopped at indicated time points with equal volume of 80% formamide, 25 mM EDTA. A. Reaction products without dNTP were resolved on 8% urea polyacrylamide gel. B. Reaction products with 5 nM dATP were resolved on 8% urea polyacrylamide gel. Position of the 25 nt primer is indicated. C. Exonuclease activity was calculated as the amount of released dNMP from the reaction products. The proportional amount of the products was calculated from the intensity of the signal and the amount of the dNMP released during the reaction, taking into account the size of each of the exonuclease products. The amount of released dNMP was plotted against time. Filled square – reaction in the absence of the dNTP, empty circle – reaction in the presence of 5 nM dATP.

Next, we analyzed the exonuclease activity of FL-Mip1, Mip1Δ175 and Mip1Δ216 in the presence of 5 nM dATP, enabling single correct nucleotide incorporation by the DNA polymerase (Figure 7). When all four dNTPs were omitted from the reaction, FL-Mip1, Mip1Δ175 and Mip1Δ216 displayed fairly similar exonuclease activity (Figure 7A). Addition of dATP shifted the balance of the reaction towards polymerization and a substantial part of the primer was elongated to 26 nt (Figure 7B). The exonuclease activity was calculated as the amount of released dNMP as described in Materials and Methods (Figure 7C). Upon addition of dATP, the exonuclease activity of FL-Mip1 and Mip1Δ175 was substantially reduced, indicating a switch of the activity towards polymerisation (Figure 7C). The exonucleolytic hydrolysis by Mip1Δ216, on the other hand, was not affected by the addition of dATP, as compared to FL-Mip1 and Mip1Δ175 (Figure 7C).

Our data shows that under limiting dNTP concentration or in the absence of the correct nucleotide to be incorporated, the exo/pol balance of the Mip1Δ216 mutant is strongly shifted towards exonucleolytic hydrolysis instead of DNA polymerization. Preference for exonuclease activity could cause inefficient mtDNA replication and be the reason for high frequency of *petite* formation observed for the *mip1Δ216 S. cerevisiae* strain [16]. Disturbed exo/pol balance of the Mip1Δ216 mutant indicates that the CTE could be involved in stabilization of the primer end at the polymerase active site.

### CTE is required for efficient strand displacement activity

**Figure 8:**
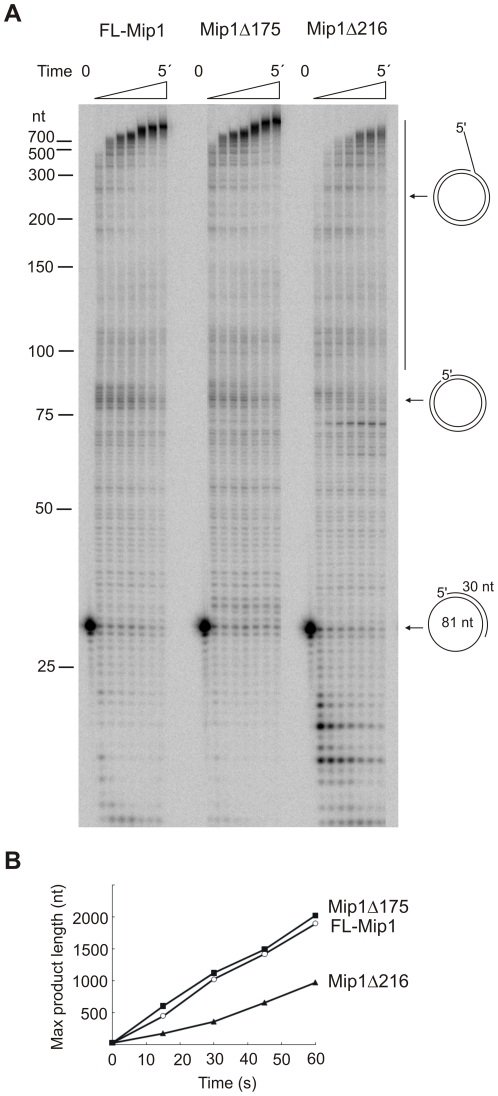
Strand displacement activity of Mip1 and C-terminal deletion mutants. Strand displacement activity was measured on an 81 nt minicircle DNA substrate singly primed with a 30 nt primer. The reaction was performed with 12 nM DNA polymerase, 20 nM minicircle substrate and 100 µM dNTP. The reaction was carried out at 30°C and stopped after 0 s, 15 s, 30 s, 45 s, 1 min, 2 min, 3 min or 5 min. A. Reaction products were resolved on denaturating 8% urea polyacrylamide gel. The position of the reaction substrate, product elongated to the full-length minicircle size and the products of strand-displacement reaction are indicated with arrows. B. Maximum product length was plotted against time. FL-Mip1 – empty circle, Mip1Δ175 –filled square, Mip1Δ216 – filled triangle.

Mip1 has been previously shown to perform extensive strand displacement DNA synthesis [15]. To assess the requirement of the CTE in DNA synthesis on double-stranded DNA substrates, we compared the strand displacement activity of FL-Mip1, Mip1Δ175 and Mip1Δ216 on a 81 nt minicircle DNA template (Figure 8). Both Mip1Δ175 and Mip1Δ216 mutants were able to displace complementary DNA strand during synthesis, however Mip1Δ216 displayed substantially reduced substrate elongation activity on minicircle DNA (Figure 8A). FL-Mip1 and Mip1Δ175 displayed similar strand displacement synthesis rates of 31 and 32 nt/s, respectively (Figure 8B). A considerably lower fraction of the primed DNA template was subjected to elongation by Mip1Δ216 and a larger fraction of the primer was subjected to degradation, confirming the disturbed exo/pol balance (Figure 8A). More importantly, the ability of Mip1Δ216 to displace the DNA strand was clearly impaired. Significant accumulation of a ∼70 nt reaction product could be detected, possibly indicating that the DNA polymerase stalled upon reaching the double-stranded substrate region (Figure 8A). The rate of Mip1Δ216 strand displacement DNA synthesis was 16 nt/s, which is significantly lower than that of FL-Mip1. These results indicate that strand displacement DNA synthesis by the Mip1Δ216 mutant is less efficient than synthesis by FL-Mip1, suggesting that the CTE is required for DNA synthesis on double-stranded templates.

## Discussion

Mitochondrial DNA polymerases in different yeast species contain a unique C-terminal extension not present in their homologues from higher eukaryotes. The CTE has been shown to be essential for mtDNA maintenance in *S. cerevisiae* [16]. Furthermore, yeast pol γ has several distinctive biochemical differences compared to metazoan mtDNA polymerases. *S. cerevisiae* Mip1 is a highly processive single subunit enzyme that apparently does not associate with additional factors to stimulate its activity, and it is able to perform extensive strand displacement DNA synthesis [15], [21]. Here we have investigated the importance of the CTE region for the biochemical functions of Mip1 polymerase.

Defined as the region of yeast pol γ starting from 16 amino acids past the conserved motif γ6, the CTE varies significantly in length among different yeast species [16]. Mitochondrial DNA polymerases from *Saccharomyces* species including *S. cerevisiae* have mostly long CTEs of approximately 280 amino acids (Figure 1A). In contrast, *Pichia* species tend to have short CTEs, such as the 68 amino acids long CTE in *Pichia pastoris*. In *Candida* species the length of the CTE varies from medium (*C. albicans*, 178 aa) to very long (*C. parapsilosis*, 356 aa). Accordingly multiple sequence alignments of the CTE region reveal a gradient of sequence homology with a more conserved part of the CTE close to the polymerase domain (Figure 1B) [16]. Based on homology, the CTE could be subdivided into three regions: highly conserved (residues 975–1038 of *S. cerevisiae* Mip1), moderately conserved (residues 1039–1079) and poorly conserved (residues 1080–1254) [16]. Interestingly, short CTEs of *Saccharomycetes* pol γ tend to end just after the highly conserved part of the region (Figure 1B).

To investigate the biochemical function of the CTE, the C-terminal deletion mutants of *S. cerevisiae* pol γ Mip1Δ279, Mip1Δ216 and Mip1Δ175 were purified, and their activity was evaluated *in vitro*. Our data indicate that the highly conserved part of the CTE is absolutely required for polymerase activity (Figure 2B, 3, 6A). The mutant *S. cerevisiae* strain that carries the *mip1Δ279* allele displays respiratory deficiency as a consequence of mitochondrial genome loss [16]. Our data reveal that Mip1Δ279 is absolutely devoid of polymerase activity, explaining the rho^0^ *in vivo* phenotype. However, we show that Mip1Δ279 binds DNA substrate and displays clearly detectable exonuclease activity (Figure 3A, 5A, 6A). This indicates that a complete deletion of the CTE mostly affects the polymerase activity. The highly conserved part of the CTE could therefore be an essential part of the DNA polymerase domain required for its activity. Optimal positioning of the primer and template strands at the polymerase active site is achieved through conformational change in the fingers subdomain [23], [34]. Yeast pol γ has a ∼40 amino acid gap within the fingers subdomain compared to its human counterpart [5], [23], [34]. Thus, the highly conserved part of the CTE could possibly function to support the fingers subdomain in positioning the template at the polymerase active site. This could be the cause of the loss of the polymerase activity in Mip1 mutants carrying a complete deletion of the CTE. Alternatively, the deletion of the 279 C-terminal amino acids could affect folding of the polymerase active site, leading to the observed loss of the DNA polymerase activity.

The Mip1Δ216 mutant retains only the highly conserved polymerase proximal part of the extension. *In vivo*, the deletion of 216 amino acids from the Mip1 C-terminus causes a strong respiratory deficient phenotype [16]. The mutant *mip1Δ216 S. cerevisiae* strain rapidly looses mtDNA when grown on glucose. Nevertheless, this mutant is able to maintain the mtDNA when grown on a non-fermentable carbon source where maintenance of the functional respiratory chain is essential for cell viability. Under these conditions the *mip1Δ216* strain displayed reduced levels of total mtDNA (∼3 times) and increased doubling time (∼2 times) as compared to wild-type [16].

Here we show that Mip1Δ216 is defective in balancing its polymerase and 3′-5′ exonuclease catalytic activities. The exo/pol balance was strongly shifted towards exonuclease activity when dNTP concentration was lowered to high nanomolar levels characteristic for mitochondria (Figure 6) [35]. Exonuclease activity of Mip1Δ216 was also dominant over polymerase activity when further synthesis was restricted due to the absence of the correctly pairing nucleotide (Figure 7). However the change in the exo/pol balance was not due to the changes in the nucleotide incorporation activity (k_pol_, Figure 3) or nucleotide excision acivity (Figure 7A).

Coordination of the polymerization and exonucleolysis is important for a proofreading DNA polymerase in order to maintain high fidelity during processive DNA replication. Several mutations have been associated with disturbed exo/pol balance in the proofreading DNA polymerases [23], [36]–[38]. These mutations tend to localize to different parts of the protein including exonuclease and polymerase domains as well as the linker domain connecting two catalytic functions. However several structural elements responsible for the control of the balance between synthesis and excision are localized in close proximity on the three-dimensional structure [36]. Our results suggest that C-terminal extension of yeast mtDNA polymerase participates in determination of the exo/pol balance, most likely by stabilizing substrate primer end at the polymerase active site.

*In vivo* exo/pol balance mutations of Mip1 are often associated with yeast hypermutator or antimutator phenotypes causing increased or reduced fidelity of DNA replication that is stimulated in the mismatch repair deficient background (*msh1-1*) [23], [36]. Interestingly prematurely terminated Mip1 with CTE deletion of 187 aa was recently identified as a strong antimutator allele [36]. This phenotype correlates well with our biochemical analysis of Mip1Δ216 mutant protein that also demonstrates shifted exo/pol balance.

Shift in the exo/pol balance often affects the efficiency of the polymerization resulting in less total amount of the DNA synthesis [23], [36]. Accordingly we found that Mip1Δ216 was less efficient in primer elongation than FL-Mip1 causing significantly lower amount of the substrate subjected to polymerization (Figure 6, 8A). However in the contrary to the other exo/pol balance mutants [23], [36], Mip1 CTE deletion did not reduce its DNA synthesis processivity and DNA substrate binding affinity (Figure 4, 5). We propose that despite stronger complex formation with DNA substrate, Mip1Δ216 mutant preferentially positions the primer end in the exonuclease active site thus causing reduced amount of substrate subjected to elongation. Increased preference of the exonuclease over polymerase activity could explain the loss of mtDNA in the *mip1Δ216* mutant yeast strain during growth on glucose containing media and reduction of mtDNA levels when grown on non-fermentable carbon source.

Our results also indicate that high processivity of Mip1 is not established by the positively charged CTE as it was previously assumed. In support of that several mutations in the exonuclease domain not affecting exonuclease activity have been shown to be detrimental for Mip1 processivity [23].

Mip1 was previously shown to displace duplex DNA upon replication [15]. The physiological importance of this activity has not been tested experimentally. However, this activity could be required for mtDNA replication *in vivo*, particularly because there is no clear evidence of a specific helicase at the yeast mitochondrial replication fork [39]. Interestingly, the Mip1Δ216 mutant also displayed reduced strand displacement activity, as the rate of primer elongation on duplex substrate was two times lower than that of FL-Mip1 (Figure 8). Mip1Δ216 had also reduced polymerization activity on gapped substrate (calf-thymus activated DNA) that could reflect its defect in displacing duplex DNA strand (Figure 2). Increased preference for exonuclease activity can affect strand-displacement activity of the DNA polymerases [40]. Thus CTE is probably not actively involved in strand separation, however by stabilizing the primer end at the polymerase site it could stimulate synthesis through double-stranded regions. Interestingly metazoan pol γ that lack the C-terminal extension are also unable to displace duplex DNA and require helicase activity for strand displacement DNA synthesis [26]. Another member of the family A DNA polymerases, the T5 DNA polymerase, is also known to loose its strand displacement activity upon the removal of its extended C-terminus [24]. Thus, importance of the extended C-terminus for strand displacement activity could be a feature shared by several DNA polymerases of family A.

The Mip1Δ175 mutant lacks a highly variable part of the CTE comprising 63% of *S. cerevisiae* Mip1 C-terminal extension. Our data showed only few minor differences between enzymatic properties of Mip1Δ175 and FL-Mip1. Accordingly, the *S. cerevisiae* strain carrying the *mip1Δ175* deletion mutant was able to maintain its mtDNA and respiratory competency *in vivo* [16]. Our data indicates that this region is not essential for polymerase function, which is in line with the high variability of this structure in different yeast species. However, this part of the CTE could provide an advantage for the polymerase during stress conditions such as high temperature, osmotic pressure or oxidative stress, allowing evolutional maintenance of this region.

In conclusion, we have demonstrated the functional importance of the C-terminal extension characteristic to mtDNA polymerases of yeast species. Our data show that complete removal of the CTE leads to the loss of polymerase dNTP incorporation activity. Partial deletion of the CTE, however, results in increased preference for exonuclease activity instead of polymerization, as well as in reduced strand displacement activity. Based on our data we propose that the unique C-terminal extension of yeast mitochondrial DNA polymerases could be involved in primer end stabilization at the polymerase active site. The CTE is an important structural component of the single subunit mitochondrial DNA polymerase, and thus further functional and structural studies could be of great interest.

## Materials and Methods

### Expression and purification of recombinant proteins

Bacterial plasmids were designed to express FL-Mip1, Mip1Δ175, Mip1Δ216 and Mip1Δ279 proteins. Recombinant proteins have a 6×His tag at the N-terminus but do not have the mitochondrial targeting sequence. The expression constructs of FL-Mip1, Mip1Δ175, Mip1Δ216 and Mip1Δ279 were made from the previously described Mip1 expression construct based on pET24d [15]. The N-terminal 6×His tag was introduced by replacing the *Nhe*I-*Afl*II fragment with a PCR product generated with the primers C1 and C2 (Table 1). The C-terminal deletions were introduced and the C-terminal His tag was removed by replacing the *Bpu*1102I-*Age*I fragment with a PCR fragment obtained with the primers C4, C5, C6, C7 in combination with the primer C3 (Table 1). The expression constructs were verified by sequencing.

**Table 1:** DNA oligonucleotide sequences.

Oligonucleotides used for expression construct cloning	
Oligo name	Sequence (5′-3′)
C1	GGACGCTAGC **CATCACCATCACCATCAC**AGCAGCACCAAGAAGAA TGCCGCAGA
C2	TTGCTTAAGGACTTTAAACCTGCAAACGAA
C3	CGCACCGGTACTGGGATGTGGTATTACCTA
C4	CGCAGCTGAGCTTAGTACTCTCTAGAAATAGTAATG
C5	CGCAGCTGAGCTTAGGATCCCATAATACGTACTTTA
C6	CGCAGCTGAGCTTACTCATCTTCTAGCCGATTCAC
C7	CGCAGCTGAGCTTATAGCAGTTGATTGATATCAAGC

Oligonucleotides used in activity assays	
Oligo name	Sequence (5′-3′)
USP	GTAAAACGACGGCCAGT
D45	GGACGGCATTGGATCGAGGTTGAGTTGGTTGGACGGCTGCGAGGC
D25	GCCTCGCAGCCGTCCAACCAACTCA
M81	TGAATTCTAATGTAGTATAGTAATCCGCTCTAAGCCATGCCTCGAC CGCTATAGTTTGTATCGTCACCATAACTCTGTAAC
M30	GAGCGGATTACTATACTACATTAGAATTCA

Recognition sites of the restriction endonucleases are underlined. 6×His tag coding sequence is in bold.

FL-Mip1 and C-terminal deletion mutant proteins were expressed in *E. coli* strain BL21-CodonPlus(DE3)-RIL. Cell lysate was prepared in 25 mM Hepes-Na pH 7.5, 500 mM NaCl, 10% glycerol, 1 mM PMSF as described previously [15]. Cleared cell lysate was loaded onto a HisTrap FF Crude column at 20 mM imidazole concentration using the ÄKTA Purifier (GE Healthcare). The column was washed with 150 mM imidazole and eluted with 500 mM imidazole containing buffer. For Mip1Δ279 batch purification, Ni-NTA agarose (Qiagen) was used, as the protein displayed inefficient binding to the HisTrap FF Crude column. Peak fractions containing Mip1 were pooled and loaded onto a SP HP column (GE Healthcare). The column was washed with 250 mM NaCl and eluted with a 250–1000 mM gradient of NaCl. Peak fractions were pooled and dialyzed against 25 mM Hepes-Na pH 7.5, 100 mM NaCl, 50% glycerol, 0.1 mM EDTA, 1 mM DTT. Aliquots of the purified proteins were frozen in liquid nitrogen and stored at −80°C before use in activity assays. Protein concentration was calculated from A280 absorbance. Molar absorption coefficient values for each protein (FL-Mip1 – 180.2, Mip1Δ175 – 169.6, Mip1Δ216 – 163.7, Mip1Δ279 - 154.7 M^−1^ cm^−1^) were calculated as previously described [41].

### Polymerase specific activity

Polymerase specific activity was measured by incorporation of [α -^32^P]-dCTP (Perkin Elmer) into calf thymus activated DNA (Sigma) as described previously [15]. The amount of incorporated [α -^32^P]-dCTP was plotted against protein concentration and the specific activity was calculated from the slope of the resulting graph.

### The gel shift mobility assay

Gel shift mobility assay was performed with the substrate 45/25 consisting of a 45 nt template oligonucleotide annealed to a 25 nt radiolabeled primer oligonucleotide (D45 and D25 respectively; Table 1). The following reaction mixture was incubated for 2 min at 0°C: 1 nM substrate 45/25, 0.2–12.5 nM DNA polymerase, 20 mM Tris-HCl pH 8.0, 40 mM KCl, 10% glycerol, 0.1 mg/ml BSA, 2 mM DTT. Samples were analyzed on a native 25 mM Tris, 190 mM glycine 8% polyacrylamide gel (37.5∶1 acrylamide∶bisacrylamide ratio) at 8 V/cm at 4°C. The dissociation constant K_D_ was estimated from the logarithmic binding curve as the concentration of the polymerase required for binding 50% of the substrate. Data from three independent experiments were used to establish the K_D_ value with standard deviation.

### Catalytic activity assays

DNA substrates were prepared by radiolabelling the 5′end of the primer oligonucleotide with T4 polynucleotide kinase (Fermentas) and [γ-^32^P]-dATP (Perkin Elmer). The radiolabeled primer oligonucleotide was annealed to the template oligonucleotide by slow cooling from 70°C to room temperature. The singly primed substrate was purified using the QIAquick PCR purification Kit (Qiagen). Polymerase catalytic assays were performed in a standard buffer of 20 mM Tris-HCl pH 8.0, 40 mM KCl, 2 mM DTT, 0.5 mg/ml BSA and 10 mM MgCl_2_ with dNTP, enzyme and substrate concentrations as indicated. Reactions were started by the addition of dNTP and MgCl_2_ to the enzyme/substrate mixture and were performed at 30°C. Reactions were stopped with equal volume of 80% deionized formamide, 25 mM EDTA, heat denaturated and resolved on 8% urea polyacrylamide gel if not stated otherwise. DNA markers were prepared from GeneRuler 1 kb and Low Range DNA ladders (Fermentas) by radiolabelling with T4 polynucleotide kinase and [γ-^32^P]-dATP. After removal of the free [γ-^32^P]-dATP with QIAquick Nucleotide Removal Kit (Qiagen), radiolabeled markers were denaturated prior to loading on the gel at the same conditions as the activity assay samples. The gels were dried under a vacuum and exposed to a PhosphoImager screen. Data were analyzed using the ImageQuant TL 2005 software package (GE Healthcare).

#### The polymerase activity assay

The polymerase activity assay was performed with 4 nM M13mp18 circular ssDNA singly primed with the radiolabeled oligonucleotide USP (Table 1), 40 nM DNA polymerase and 100 µM dNTP. The longest product with the signal exceeding the background level at least three times was used to estimate the k_pol_ value.

#### The processivity assay

The processivity assay was performed with M13 ssDNA singly primed with the radiolabeled oligonucleotide USP. 4 nM DNA polymerase was preincubated with 4 nM substrate and the reaction was started by the addition of 100 µM dNTP, 10 mM MgCl_2_ and 1 mg/ml calf thymus activated DNA. The reaction was stopped by the addition of an equal volume of 0.5 mg/ml Proteinase K, 1% SDS and 20 mM EDTA. Reaction products were phenol/chloroform extracted, ethanol precipitated and separated on a 0.8% alkaline agarose gel. The processivity value was calculated using weighted mean values based on product intensity (i) and length (l).
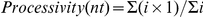
Data from three independent experiments were used to establish the average processivity with standard deviation. For each reaction, a competitor DNA efficiency control was performed where calf thymus activated DNA was added to the reaction along with the radiolabeled substrate.

#### The polymerase and exonuclease balance assay

The polymerase and exonuclease balance assay was performed with the substrate 45/25. Standard buffer conditions were used with 4 nM DNA polymerase and 10 nM substrate. The final dNTP concentration was varied from 1 to 125 nM. The reaction was stopped after 5 min with an equal volume of formamide buffer. The percentage of polymerization products among all reaction products was quantified and plotted against dNTP concentration.

#### The exonuclease assay

The exonuclease assay was performed with the substrate 45/25. Standard buffer conditions were used with 4 nM DNA polymerase and 2 nM substrate. The assay was performed in the absence of dNTP or in the presence of 5 nM dATP. Released dNMP was calculated for each timepoint considering the intensity (i) and length (l) of each exonuclease product produced at this point. The relative amount of dNMP released was then normalized to molar amount from initial substrate concentration (20 fmol of substrate per 10 µl reaction).
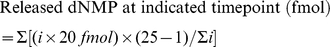



#### Strand displacement activity

Strand displacement activity was measured on the 81 nt minicircle DNA substrate singly primed with a 30 nt primer (M81 and M30 respectively, Table 1). The 81 nt minicircle DNA substrate was prepared as described previously [15]. The reaction was performed under standard conditions with 12 nM DNA polymerase, 20 nM minicircle substrate and 100 µM dNTP. Maximum product length was determined as the longest product with the signal exceeding background levels at least two times.
